# Guilt is associated with acute stress symptoms in children after road traffic accidents

**DOI:** 10.3402/ejpt.v6.29074

**Published:** 2015-10-28

**Authors:** Ann-Christin Haag, Daniel Zehnder, Markus A. Landolt

**Affiliations:** 1 Department of Psychosomatics and Psychiatry, University Children's Hospital, Zurich, Switzerland; 2 Children's Research Center, University Children's Hospital, Zurich, Switzerland; 3 Clienia Schloessli AG, Oetwil am See, Switzerland; 4 Department of Child and Adolescent Health Psychology, Institute of Psychology, University of Zurich, Switzerland

**Keywords:** Trauma, cognitive appraisals, childhood, injury

## Abstract

**Background:**

Although previous research has consistently found considerable rates of acute stress disorder (ASD) in children with accidental injuries, knowledge about determinants of ASD remains incomplete. Guilt is a common reaction among children after a traumatic event and has been shown to contribute to posttraumatic stress disorder. However, its relationship to ASD has never been examined.

**Objective:**

This study assessed the prevalence of ASD in children and adolescents following road traffic accidents (RTAs). Moreover, the association between peritraumatic guilt and ASD was investigated relying on current cognitive theories of posttraumatic stress and controlling for female sex, age, socioeconomic status (SES), injury severity, inpatient treatment, pretrauma psychopathology, and maternal posttraumatic stress symptoms (PTSS).

**Methods:**

One hundred and one children and adolescents (aged 7–16 years) were assessed by means of a clinical interview approximately 10 days after an RTA. Mothers were assessed by questionnaires.

**Results:**

Three participants (3.0%) met diagnostic criteria for full ASD according to DSM-IV, and 17 (16.8%) for subsyndromal ASD. In a multivariate regression model, guilt was found to be a significant predictor of ASD severity. Female sex, outpatient treatment, and maternal PTSS also predicted ASD severity. Child age, SES, injury severity, and pretraumatic child psychopathology were not related to ASD severity.

**Conclusions:**

Future research should examine the association between peritraumatic guilt and acute stress symptoms in more detail. Moreover, guilt appraisals in the acute phase after an accident might be a relevant target for clinical attention.

With 186,300 children dying from road traffic accidents (RTAs) worldwide each year and many more injured, they represent particularly vulnerable road users (World Health Organization, 2015). Because many RTAs meet criteria for a traumatic event, as defined by the *Diagnostic and Statistical Manual of Mental Disorders*—Fifth Edition (DSM-5; American Psychiatric Association [APA], 2013), acute stress disorder (ASD) is a common subsequent psychological reaction in children and adolescents (Ellis, Stores, & Mayou, 1998). ASD is defined *via* five symptom categories: intrusion, negative mood, dissociation, avoidance, and hyperarousal (APA, 2013).

Previous studies among school-aged victims of RTAs that utilized DSM-IV diagnostic criteria (APA, 1994) have revealed ASD prevalence rates between 1.6 and 15.0% (*Mdn*=8.0%; Bryant, Mayou, Wiggs, Ehlers, & Stores, 2004; Kassam-Adams & Winston, 2004; Meiser-Stedman et al., 2007; Winston, Baxt, Kassam-Adams, Elliott, & Kallan, 2005; Winston et al., 2002). These widely divergent rates are likely related to methodological issues, such as small and heterogeneous samples and the lack of any standardized assessment of ASD criteria.

Although numerous studies have been published on risk factors for posttraumatic stress disorder (PTSD) in children and adolescents (for a review, see Trickey, Siddaway, Meiser-Stedman, Serpell, & Field, 2012), there is little research specifically on determinants of ASD. Published studies have generated inconsistent results regarding trauma- and individual-related variables (e.g., Di Gallo, Barton, & Parry-Jones, 1997; Kassam-Adams & Winston, 2004; Meiser-Stedman et al., 2007). Consistent associations have been found between children's acute stress symptoms and parental distress (Daviss et al., 2000; Saxe et al., 2005). However, to the best of our knowledge, only one previous study has examined the association between peritrauma variables and DSM-IV (APA, 1994)-based ASD in school-aged children and adolescents following RTAs *via* a multivariate predictive model. This study identified injury severity and receiving medical care as significant predictors of ASD symptoms (Winston et al., 2005).

Compendiously, previous research on determinants of ASD has not examined the relationship between peritraumatic guilt for a traumatic event and ASD in children and adolescents. However, with a prevalence rate of 33% among school-aged children, guilt is a common reaction after they experience a traumatic event (Fletcher, 2003). In addition, guilt appraisals usually begin in the early aftermath of a traumatic event and, therefore, could influence acute stress symptoms.

According to Wilson, Droždek, and Turkovic (2006), guilt is self-recrimination for failed personal enactments. Kubany and Watson's concept of posttraumatic guilt (2003) posits two determinants—distress and cognitions—and has most recently gained empirical support (Browne, Trim, Myers, & Norman, 2015). Guilt is a negative appraisal and can thus have detrimental effects upon posttraumatic adjustment (Foa, Ehlers, Clark, Tolin, & Orsillo, 1999). The widely recognized cognitive model of PTSD by Ehlers and Clark (2000) describes how negative appraisals of a traumatic event, like guilt, lead to posttraumatic stress because they maintain a sense of threat. This hinders the individuals from properly processing the traumatic event. There is also evidence that this model can theoretically (Meiser-Stedman, 2002) and empirically be applied to children and adolescents after RTAs (Ehlers, Mayou, & Bryant, 2003).

Notably, several studies on accidentally injured children and adolescents have identified an association between posttraumatic stress and negative appraisals (Bryant, Salmon, Sinclair, & Davidson, 2007; Hitchcock, Ellis, Williamson, & Nixon, 2015; Meiser-Stedman, Dalgleish, Glucksman, Yule, & Smith, 2009) and trauma-related guilt in particular (Greenberg & Keane, 2001). Moreover, both cross-sectional and longitudinal studies involving other childhood-trauma samples have consistently revealed the negative effect of guilt on posttraumatic stress symptoms (PTSS; e.g., Kletter, Weems, & Carrion, 2009; Melville, Kellogg, Perez, & Lukefahr, 2014; Punamäki, Palosaari, Diab, Peltonen, & Qouta, 2014; Stotz, Elbert, Müller, & Schauer, 2015). Based on this research, guilt—specifically, self-blame for a traumatic event—now is listed as a symptom of PTSD in DSM-5 (APA, 2013).

To summarize, there is a shortage of studies that examine the prevalence and determinants of ASD in children and adolescents after RTAs. Specifically, studies investigating the role of peritraumatic guilt in ASD are completely lacking. The aims of the present study were, therefore, twofold. First, we aimed to assess the prevalence of ASD in school-aged children and adolescents after RTAs. Based on previous findings, we expected that the prevalence of ASD would be in the range of 6–10%. Second, we sought to examine the association between guilt, specifically self-blame for a traumatic event, and the severity of ASD symptoms, while controlling for demographic, medical, and individual- and trauma-related variables (female sex, age, socioeconomic status (SES), injury severity, inpatient treatment, pretraumatic psychopathology, and maternal PTSS). Based on the cognitive model of PTSD by Ehlers and Clark (2000) and other previous research, we hypothesized that guilt would have a significant positive association with ASD severity.

## Methods

### Participants

Participants consisted of children and adolescents ranging from 7 to 16 years who had experienced an RTA as well as their mothers. Children and adolescents were eligible for participation if the following inclusion criteria were met: 1) medical treatment (inpatient or outpatient) at University Children's Hospital, Zurich immediately after an RTA; 2) fluency in German; 3) no severe head injury (Glasgow Coma Scale [GCS] >11); and 4) no previous evidence of cognitive impairment (according to medical records). Participants were recruited continuously over a period of 3 years. Families were contacted within the first week of their child's accident. Out of 139 children and adolescents who met the inclusion criteria, 38 refused to participate, the primary reasons being lack of interest or time. The final study sample, therefore, consisted of 101 children or adolescents and their mothers (*N*=93). Non-participants and participants did not differ significantly in terms of their age (*t*=−0.36, *p*=0.72), sex (*χ* ^2^=2.96, *p*=0.09), nationality (*χ* ^2^=6.19, *p*=0.40), severity of injuries as defined below (*U*=1,531.50, *p*=0.07; non-participants: *M*=4.89, *SD*=5.23, *Mdn=*3.00) or whether they had experienced the RTA as a passenger, pedestrian, cyclist, or other (*χ* ^2^=3.00, *p*=0.56).

### Procedures

This study was embedded within a randomized controlled trial (RCT) evaluating the effectiveness of an early psychological intervention for school-aged children and adolescents after an RTA (Zehnder, Meuli, & Landolt, 2010). In this study, we present extended baseline data collected approximately 10 days after the accident, before allocation of participants to the two treatment arms of the RCT. In accordance with ethics standards, written informed consent was obtained from all parents and all children above the age of 12. Children and adolescents were assessed by means of a standardized face-to-face interview (30–45 min), either at the participant's home or at the hospital. Mothers were assessed at the same time by means of a written questionnaire. Medical data were obtained from patient records and attending physicians.

### Measures

#### Child acute stress disorder (ASD)

Symptoms of accident-related ASD in children and adolescents were assessed using the IBS-A-KJ (“Interview zur Akuten Belastungsstörung bei Kindern und Jugendlichen”; Steil & Füchsel, 2006), a standardized clinical interview based on DSM-IV criteria (APA, 2000). Using a five-point Likert scale, the frequency and intensity of all ASD symptoms (clusters B–E: dissociation, intrusion, avoidance, and hyperarousal) were assessed. A symptom was considered present if the frequency score was at least “1” (“rarely”) and the intensity was rated as at least “2” (“somewhat”). In addition, functional impairment was assessed and an ASD diagnosis assigned according to the DSM-IV (APA, 2000). Subsyndromal ASD was diagnosed if criteria for at least three of the four symptom clusters were met (Bryant et al., 2007). For each of the four symptom clusters, a severity score was derived by summing up both the frequency and intensity items. A total ASD severity score was calculated by adding up all symptom items. The IBS-A-KJ is considered a reliable and valid measure of ASD in children and adolescents (Steil & Füchsel, 2006). In this study, internal consistency of the IBS-A-KJ total ASD severity score was found to be good (Cronbach's α=0.86).

#### Guilt

Guilt—i.e., self-blame for the accident—was assessed during the interview with the child by means of a single question: “Do you feel guilty for causing the accident?” Response options indicating the frequency of feelings of guilt were provided on a three-point Likert scale (“never”, “sometimes”, “frequently”).

#### Pretrauma psychopathology

Pretrauma psychopathology was assessed by asking the mother if her child had ever received psychological treatment before the accident.

#### Injury severity

Injury severity was rated by a physician using the Modified Injury Severity Scale (MISS; Mayer, Matlak, Johnson, & Walker, 1980). This highly reliable and widely used instrument measures the severity of injuries in different bodily systems. Severity scores range from 1 (no injury) to 75 (a life-threatening injury) with scores greater than 25 indicating a severe injury.

#### Inpatient treatment

Information about admission for inpatient treatment—i.e., spending at least one night at hospital—was retrieved from the patients’ medical records.

#### Loss of consciousness

Whether initial loss of consciousness occurred after the accident was retrieved from the patients’ medical records.

#### Parental posttraumatic stress

To assess PTSS in mothers, the German version (Ehlers, Steil, Winter, & Foa, 1996) of the Posttraumatic Diagnostic Scale (PDS; Foa, 1995) was used. The PDS provides both a score for PTSD severity and a categorical PTSD diagnosis—here related to the child's RTA—as per the DSM-IV (APA, 1994). In this study, the sum of all 17 symptom items (criteria B–D) was calculated and used as a measure of PTSD severity, with scores ranging from 0 to 51 and higher scores indicating more severe PTSD symptoms. Mothers rated the frequency of each symptom on a four-point Likert scale. The internal consistency of the scale in this study was excellent (Cronbach's α=0.93) and almost identical to that of the original version (Foa, Cashman, Jaycox, & Perry, 1997).

#### Socioeconomic status (SES)

SES was calculated *via* the summation of two ratings: 1) paternal occupation and 2) maternal education. Each rating was scored 1–6, with summation scores ranging from 2 to 12 points. Three social classes were defined: SES scores 2–5, lower class; SES scores 6–9, middle class; and SES scores 10–12, upper class. This measure has been shown to be a reliable and valid indicator of SES in Switzerland (Landolt, Vollrath, Ribi, Gnehm, & Sennhauser, 2003).

#### Demographics

Demographic information (sex, age, nationality) was obtained from interviews with the children and adolescents, as well as from questionnaires completed by the parents.

### Statistical analysis

Data were analyzed using SPSS statistical software for Windows, version 20 (SPSS Inc., Chicago, IL, USA). Comparisons between participants and non-participants were performed with Student's *t*-tests for continuous data, *χ* ^2^ tests for categorical variables, and Mann–Whitney *U* tests to assess differences in variables with non-normal distributions. Kolmogorov–Smirnov goodness-of-fit tests were performed to test for normality of the dependent variable (ASD severity score). Because the variable was not normally distributed (K-S-Z=1.60, *p*=0.01), a square-root transformation was performed to achieve normality (K-S-Z=0.09, *p*=0.07). ASD severity scores per cluster were also square-root transformed. However, they did not achieve normality. Bivariate associations of studied variables were examined by means of Spearman correlation coefficients due to the non-normal distribution of many of the variables. Multiple linear regression analysis was performed to analyze guilt as a predictor variable of ASD, while controlling for other independent variables. Likewise, the four ASD symptom clusters were analyzed in separate multivariate predictive models. Based on the literature and the strength of bivariate associations with ASD severity, the following independent variables were chosen for entry into the model: guilt appraisal, sex, age, SES, injury severity, inpatient treatment, pretrauma psychopathology of the participating child or adolescent, and maternal PTSS.

## Results

### Sample characteristics

The mean age of the child and adolescent participants was *M*=11.55 years (*SD*=2.70, *Min*=7.08, *Max*=16.33). Fifty-nine (58.4%) were male. There were more Swiss families (*n*=75, 74.3%) than non-Swiss families. Children were involved in an RTA either as a passenger (car 15.8%, motorcycle 6.9%), pedestrian (33.7%), cyclist (27.7%), or other (15.8%). The vast majority of the mothers were not involved in the RTA (86.1%); some witnessed the accident (12.9%), whereas one was injured in the same accident (1.0%). Considering the possible range of injury severity (1–75), the MISS was low in most subjects, as indicated by a mean *M*=5.92 (*SD*=4.96, *Mdn*=5.00). Sixteen patients (15.8%) experienced loss of consciousness. Twelve (12.0%) suffered from some mild traumatic brain injury (*n*=2 with a GCS score of 12; *n*=3 with GCS of 13; *n*=7 with GCS of 14). The majority of the subjects were hospitalized in the aftermath of the accident (*n*=62, 61.4%) and most had not attended psychotherapy prior to the accident (*n*=81, 80.2%). The mean severity of PTSD symptoms (*M*=9.75 out of 51, *SD*=9.76) among mothers was rather low. Nearly half of the subjects were of upper SES (*n*=46, 45.5%), followed by 36 (35.6%) middle class and 11 lower class (10.9%). The SES of eight families was unknown (7.9%). Fifteen children or adolescents indicated frequent appraisals of guilt (14.9%), whereas 49 (48.5%) reported having experienced them sometimes, and 37 (36.6%) never.

### Prevalence of ASD

Table 1 presents descriptive statistics for ASD symptoms and diagnosis. Three (3.0%) participants met full criteria for ASD, as per the DSM-IV (APA, 2000), whereas 17 (16.8%) met criteria for subsyndromal ASD. About half of the subjects met criteria for intrusion and hyperarousal clusters, whereas roughly 20% met those for avoidance, and only approximately 13% met those for dissociation.

**Table 1:** Descriptive statistics for ASD symptoms and diagnosis

*Dissociation* (cluster B)	
% meeting DSM-IV criteria	12.9
Mean number of symptoms (*SD*)	0.87 (1.18)
Mean symptom severity (*SD*)	6.63 (5.88)
*Intrusion* (cluster C)	
% meeting DSM-IV criteria	50.5
Mean number of symptoms (*SD*)	1.27 (1.58)
Mean symptom severity (*SD*)	8.61 (8.29)
*Avoidance* (cluster D)	
% meeting DSM-IV criteria	20.8
Mean number of symptoms (*SD*)	0.21 (1.25)
Mean symptom severity (*SD*)	1.79 (2.30)
*Hyperarousal* (cluster E)	
% meeting DSM-IV crieria	51.5
Mean number of symptoms (*SD*)	1.00 (1.25)
Mean symptom severity (*SD*)	7.29 (7.30)
*All four symptom clusters*	
% meeting DSM-IV criteria for full ASD	3.0
% meeting DSM-IV criteria for subsyndromal ASD	16.8
Mean ASD total symptom severity (*SD*)	24.33 (19.76)

*N*=101. ASD, acute stress disorder; DSM-IV, Diagnostic and Statistical Manual of Mental Disorders (APA, 2000).

### Associations between acute stress symptomatology and study variables

Spearman correlation coefficients for study variables and ASD severity are presented in Table 2. ASD severity was significantly correlated with female sex, SES, and maternal PTSS. Table 3 summarizes statistics for the multiple regression model with total ASD severity as the dependent variable. Guilt, female sex, and maternal PTSS were found to be significant predictors of ASD severity. Moreover, having been treated as an inpatient predicted less severe ASD. Age, SES, injury severity, and pretrauma psychopathology were not found to be predictive of ASD severity. Overall, the model was significant at a *p*<0.001 level, with the predictor variables accounting for 36% of the variance in the child's ASD severity.

**Table 2:** Summary of intercorrelations for independent variables and ASD severity

	*1*	*2*	*3*	*4*	*5*	*6*	*7*	*8*	*9*
1. Female sex	–								
2. Child age	−0.03	–							
3. SES	−0.10	0.01	–						
4. Injury severity score	−0.11	0.06	0.03	–					
5. Inpatient treatment	−0.07	0.05	−0.06	0.67**	–				
6. Loss of consciousness	0.02	0.06	−0.07	0.35**	0.34**	–			
7. Guilt	−0.05	0.21*	0.11	0.00	−0.02	0.15	–		
8. Pretrauma psychopathology	0.06	0.12	0.10	−0.19	−0.20	−0.04	0.05	–	
9. PTSS mother	0.15	−0.08	−0.08	0.04	0.18	0.33**	−0.03	0.22*	
10. Child ASD severity	0.28**	0.17	−0.31**	−0.04	−0.10	0.13	0.15	0.13	0.36**

Spearman correlation coefficients. *N*=84–101. ASD, acute stress disorder; SES, Socioeconomic status; PTSS, posttraumatic stress symptoms.

* *p*<0.05.

** *p*<0.01.

**Table 3:** Summary of multiple regression analysis with ASD severity as dependent variable

	*B*	*SE B*	β	*p*
Female sex	0.77	0.37	0.19	0.04
Child age	0.09	0.07	0.12	0.20
SES	−0.13	0.08	−0.16	0.10
Injury severity score	0.00	0.04	0.00	0.99
Inpatient treatment	−0.95	0.42	−0.23	0.03
Guilt	0.63	0.26	0.22	0.02
Pretrauma psychopathology	−0.19	0.38	−0.05	0.61
PTSS mother	0.10	0.02	0.49	0.00

*N*=82. ASD, acute stress disorder; *SE B*, standard error of the regression coefficient; SES, Socioeconomic status; PTSS, posttraumatic stress symptoms. *R* ^2^=0.42, *R* ^2^ adjusted=0.36, *F*(8)=6.75, *p*<0.001.

In four separate multiple regression models for each ASD symptom cluster (Table 4) the same predictors were identified for clusters C (intrusion) and D (avoidance) as for the total ASD severity. Guilt reached borderline significance as a predictor for cluster B (dissociation, *p*=0.07), but was not significant with regard to cluster E (hyperarousal, *p*=0.13).

**Table 4:** Summary of multiple regression analyses with ASD severity clusters B–E as dependent variables

	Cluster B (dissociation)				Cluster C (intrusion)				Cluster D (avoidance)				Cluster E (hyperarousal)			
				
	*B*	*SE B*	β	*p*	*B*	*SE B*	β	*p*	*B*	*SE B*	β	*p*	*B*	*SE B*	β	*p*
Female sex	0.36	0.22	0.16	0.11	0.68	0.29	0.22	0.02	0.27	0.17	0.13	0.12	0.35	0.31	0.11	0.26
Child age	0.08	0.04	0.18	0.08	0.07	0.06	0.12	0.21	−0.12	0.03	−0.31	0.00	0.13	0.06	0.22	0.03
SES	−0.09	0.05	−0.19	0.06	−0.06	0.06	−0.10	0.33	−0.01	0.04	−0.03	0.77	−0.08	0.07	−0.12	0.25
ISS	0.06	0.03	0.26	0.02	−0.04	0.04	−0.13	0.23	−0.01	0.02	−0.04	0.67	−0.03	0.04	−0.09	0.45
Inpatient treatment	−0.41	0.26	−0.18	0.11	−0.76	0.34	−0.25	0.03	−0.54	0.20	−0.27	0.01	−0.35	0.36	−0.11	0.36
Guilt	0.29	0.16	0.18	0.07	0.45	0.21	0.21	0.03	0.41	0.12	0.30	0.00	0.34	0.22	0.16	0.13
Pretrauma psych	−0.29	0.23	−0.13	0.21	−0.16	0.30	−0.05	0.59	−0.23	0.18	−0.11	0.20	0.39	0.32	0.13	0.22
PTSS mother	0.05	0.01	0.42	0.00	0.06	0.02	0.37	0.00	0.05	0.01	0.52	0.00	0.05	0.02	0.34	0.00
	*F*(8)=5.10, *p*<0.001				*F*(8)=5.41, *p*<0.001				*F*(8)=8.92, *p*<0.001				*F*(8)=4.16, *p*<0.001			
	*R* ^2^ adj.=0.29				*R* ^2^ adj.=0.30				*R* ^2^ adj.=0.44				*R* ^2^ adj.=0.24			

*N*=82. ASD, acute stress disorder; SES, socioeconomic status; ISS, injury severity score; Pretrauma psych, pretrauma psychopathology; PTSS, posttraumatic stress symptoms; *R* ^2^ adj., *R* ^2^ adjusted. Dependent variables are not normally distributed.

## Discussion

The two primary aims of this study were to examine the prevalence of ASD among children and adolescents following an RTA and to investigate the association between guilt and ASD severity, while controlling for other independent variables.

The 3.0% prevalence of ASD in this sample is at the lower end of previously published estimates. It is considerably lower than in two previous samples (8.0%, Kassam-Adams & Winston, 2004; 15.0%, Bryant et al., 2004), but higher than the 1.6% reported by Winston et al. (2005). One reason for the rather low prevalence in our sample might be that participants were mainly from upper SES families. A higher SES usually implies more financial and social resources, which in turn can serve as protective factors in stressful situations (Trickey et al., 2012). Another reason may be that, in recent years, trauma-informed care has become more widely established at our hospital, and one could speculate that this improved care has reduced ASD rates. Finally, it is important to consider that the ASD DSM-IV criteria are probably too strict for children by requiring three dissociative symptoms to meet that criterion (Meiser-Stedman et al., 2007).

The second aim of this study was to expand upon previous research on ASD by examining its association with guilt. Consistent with our hypothesis, and in line with the cognitive model of PTSD developed by Ehlers and Clark (2000), we identified a significant concurrent relationship between guilt and ASD symptom severity, even when accounting for other relevant factors. The cognitive model of Ehlers and Clark (2000) serves as a good paradigm to show the influence of guilt on acute stress. Guilt is a negative trauma appraisal that leads to a sense of threat. This, in turn, leads to maladaptive cognitive processing styles, such as rumination and avoidance of reminders of the trauma. Thus, the proposed link to rumination and avoidance is perfectly consistent with our discovery that guilt was associated with the ASD clusters of intrusion and avoidance. Feiring and Cleland (2007) also found self-blame to be associated with intrusion, hyperarousal, and avoidance in a longitudinal study on sexually abused children. Although, to date, the role of guilt in ASD has never been studied in children and adolescents, our results are consistent with previous findings showing that guilt predicts longer term PTSS in traumatized children and adolescents (e.g., Kletter et al., 2009). Notably, our results are also in accordance with findings on the relationship between self-blame and ASD among adult burn victims (Lambert, Difede, & Contrada, 2004). Finally, guilt is consistent with core clinical symptoms of ASD (DSM-5; APA, 2013), as it may foster intrusive thoughts, negative mood, hyperarousal, and avoidant behaviours.

In this study, female sex and acute maternal PTSS also predicted child ASD severity. Our finding that girls were more vulnerable to posttraumatic stress is consistent with two previous studies among accidentally injured children (Bryant et al., 2004; Meiser-Stedman et al., 2007). The underlying mechanism behind this increased vulnerability of females is a matter of considerable controversy. In adults, Breslau and Anthony (2007) proposed an interaction among biological, cultural, and developmental factors.

Our finding that higher acute maternal PTSS predicted higher child and adolescent ASD severity is also in line with previous study results (Daviss et al., 2000; Saxe et al., 2005). One possible explanation might be that distressed mothers and fathers are less able to appropriately support their children during the difficult early posttrauma period (Stoddard et al., 2006). In the literature, there is clear evidence that the influence of parental PTSS on PTSS in their children is not only related to the acute, but also to the long-term sequelae of childhood trauma (Landolt, Ystrom, Sennhauser, Gnehm, & Vollrath, 2012).

Surprisingly, in the current study inpatient treatment was associated with less severe ASD. This is contrary to the findings of De Young, Kenardy, & Cobham (2011) regarding PTSD in young children. Reasons for this could be that children admitted to the hospital received more professional care (e.g., better availability of social workers and psychologists). Inpatient treatment could also ease the cognitive and emotional processing of the accident, because needing to communicate and thereby confront the cause, circumstances, and sequelae of the accident and the injury may lead to a better understanding of the experience.

Age, SES, injury severity, and pretrauma psychopathology were not associated with ASD in our sample. There are dissenting results reported in the literature regarding the relationship between age and ASD (e.g., Meiser-Stedman et al., 2007; Winston et al., 2005). Contrary to Trickey et al.'s (2012) meta-analysis finding of an association between PTSD and lower SES, SES was not related to acute stress in our study. One probable reason might be, as discussed above, that low SES was infrequent in our sample. Although the lack of any association between injury severity and ASD is contrary to the results of Winston et al. (2005), it is consistent with other studies on ASD in children after RTAs (e.g., Kassam-Adams & Winston, 2004; Saxe et al., 2005). Research on the importance of pretraumatic psychopathology is inconsistent as well (Daviss et al., 2000; Di Gallo et al., 1997). These inconsistencies could be due to different ages of the participants, different samples of trauma-exposed children, and inconsistent assessment of this variable, as well as the different statistical analysis methods adopted.

Although this is the first study to use a multivariate predictive model to examine guilt's role in ASD in children and adolescents after RTAs, several limitations should be mentioned. First, underrepresentation of non-Swiss and lower SES families may have biased our ASD rates to yield overly positive outcomes. Second, excluding patients with moderate-to-severe head injuries might have affected our results in an unknown way. Third, from a methodological point of view, the operationalization of guilt using a single item may be problematic, because using a single item implies some measurement error. The so-produced error variance can contribute to an underestimation of the true association between guilt and ASD. On the other hand, longer and validated measures—like the State Shame and Guilt Scale (SSGS; Marschall, Sanftner, & Tangney, 1994) or the Trauma-Related Guilt Inventory (TRGI; Kubany et al., 1996)—would have been too lengthy for the purposes of screening children in the aftermath of a traumatic event. Using one item to assess guilt is a more economic and feasible way that has been used commonly in previous studies on traumatized children (Melville et al., 2014) and adults (e.g., Bakker, Van Loey, Van Son, & Van der Heijden, 2010; Lambert et al., 2004). Fourth, the study's cross-sectional design does not allow for any causal conclusions. Therefore, we do not know whether guilt contributes to the development of ASD, ASD leads to or exacerbates guilt, or some third factor contributes to both guilt and ASD. Finally, that our multivariate model explained 36% of the variance in ASD severity indicates that other variables not assessed in the current study must also have been important. These variables might include the child's trauma history, presence or level of pain, accident-related threat appraisals, or paternal stress symptoms. Importantly, as the variables were not normally distributed, the results regarding the four ASD cluster severities must especially be interpreted with caution.

These limitations aside, the current study provides several avenues for future research. First, the validity of the single-item guilt measure, including its independence from measures of other peritraumatic factors, such as distress, should be investigated in future studies to ensure that it is guilt and not some other construct that is associated with ASD. Second, replicating our findings using more sophisticated and validated measures of guilt is required. Third, including trauma-related guilt in a concurrent screening instrument for ASD, such as the ASC-Kids (Kassam-Adams, 2006), should be considered. Fourth, as there is no evidence regarding the association of early guilt appraisals with later risk for posttraumatic stress, prospective studies are needed to examine this pathway. Furthermore, the role of guilt in different types of trauma must be investigated more thoroughly. Similarly, other forms of guilt, such as regret for not having protected others who were harmed, or survivor guilt, may also warrant investigation. Finally, another important issue for future research is to examine the association between parental and child guilt appraisals. It has been shown that parental guilt is a risk factor for parental PTSS, which in turn influences a child's posttraumatic adjustment (De Young, Hendrikz, Kenardy, Cobham, & Kimble, 2014).

With regard to clinical practice, our results suggest that addressing guilt cognitions in children and adolescents might be relevant to treating and preventing ASD symptoms. Measuring peritraumatic guilt could improve the assessment and might help to identify those children who actually develop ASD.

## Conclusions

This study provides preliminary evidence that guilt appraisals may be concurrently related to ASD in accidentally injured children and adolescents. This finding suggests that it might be important for clinicians to assess guilt appraisals at an early stage posttrauma, and to consider including cognitive strategies in early intervention protocols.
